# Cerebrolysin combined with rehabilitation promotes motor recovery in patients with severe motor impairment after stroke

**DOI:** 10.1186/s12883-016-0553-z

**Published:** 2016-03-02

**Authors:** Won Hyuk Chang, Chang-hyun Park, Deog Young Kim, Yong-Il Shin, Myoung-Hwan Ko, Ahee Lee, Shin Yi Jang, Yun-Hee Kim

**Affiliations:** Department of Physical and Rehabilitation Medicine, Center for Prevention and Rehabilitation, Heart Vascular Stroke Institute, Samsung Medical Center, Sungkyunkwan University School of Medicine, 50 Irwon-dong, Gangnam-gu, Seoul 135-710 Korea; Department and Research Institute of Rehabilitation Medicine, Yonsei University College of Medicine, Seoul, Korea; Department of Rehabilitation Medicine, Pusan National University School of Medicine, Pusan National University Yangsan Hospital, Pusan, Korea; Department of Physical Medicine and Rehabilitation, Research Institute of Clinical Medicine of Chonbuk National University, Biomedical Research Institute of Chonbuk National University Hospital, Jeonju, Korea; Department of Health Science and Technology, Department of Medical Device Management & Research, SAIHST, Sungkyunkwan University, Seoul, Korea; HVS Imaging Center, Heart Vascular Stroke Institute, Samsung Medical Center, Seoul, Korea

**Keywords:** Cerebrolysin, Imaging, Motor recovery, Rehabilitation, Stroke, Subacute therapy

## Abstract

**Background:**

Cerebrolysin is a neuropeptide preparation with neuroprotective and neurorestorative effects. Combining Cerebrolysin treatment with a standardized rehabilitation program may have a potential synergistic effect in the subacute stage of stroke. This study aims to evaluate whether Cerebrolysin provides additional motor recovery on top of rehabilitation therapy in the subacute stroke patients with moderate to severe motor impairment.

**Methods:**

This phase IV trial was designed as a prospective, multicenter, randomized, double-blind, placebo-controlled, parallel-group study. A total of 70 patients (Cerebrolysin *n* = 35, placebo *n* = 35) with moderate to severe motor function impairment were included within 7 days after stroke onset and were randomized to receive a 21-day treatment course of either Cerebrolysin or placebo, given in addition to standardized rehabilitation therapy. Assessments were performed at baseline, immediately after treatment as well as 2 and 3 months after stroke onset. The plasticity of motor system was assessed by diffusion tensor imaging and with resting state functional magnetic resonance imaging.

**Results:**

Both groups demonstrated significant improvement in motor function (*p* < 0.05); however, no significant difference was found between the two groups. In the stroke patients with severe motor impairment, the Cerebrolysin group exhibited significantly more improvement in motor function compared with the placebo group (*p* < 0.05). Effects of Cerebrolysin were demonstrated as restricted increments of corticospinal diffusivity and as recovery of the sensorimotor connectivity.

**Conclusion:**

The combination of standard rehabilitation therapy with Cerebrolysin treatment in the subacute stroke has shown additional benefit on motor recovery and plastic changes of the corticospinal tract in patients with severe motor impairment.

**Trial registration:**

NCT01996761 (November 5, 2013)

## Background

Motor impairment is a major cause of disability in activities of daily living in stroke survivors [1]. Many rehabilitation strategies attempt to enhance motor recovery in stroke patients, however, the effects are limited especially for patients with severe motor impairment [2]. Innate physiological and anatomical plasticity contributes to the substantial gains achieved in motor function after stroke, and the combination of task-specific training and general aerobic exercise is still the gold standard for post-stroke rehabilitation [3]. In particular, the subacute stage after stroke is the critical period during which the brain is most receptive to modification by rehabilitative experiences [4–6].

Cerebrolysin (EVER Neuro Pharma GmbH, Austria) is a neuropeptide preparation of low molecular-weight peptides and free amino acids with neuroprotective and neurorestorative effects [7]. Recently published trials have shown a trend for favorable outcome of Cerebrolyin in acute stroke patients [8, 9]. Specifically, Cerebrolysin has been shown to enhance neurogenesis in the dentate gyrus of the hippocampus [10], which indicates that the compound is capable of stimulating the restorative capacity of the brain after injury. Upregulation of neurogenesis occurs naturally, and plays an important role in the recovery of neurological function after an ischemic stroke [7]. Therefore, Cerebrolysin may possess the potential to accelerate this process in stroke. Cerebrolysin, furthermore, demonstrated neurotropic effects by imitating natural neurotrophic factors, in addition to the previously mentioned effects [11]. Considering that natural adaptations to injury occur rapidly and on a wide scale in the subacute stage of stroke [4–6], the subacute stage would constitute the most appropriate time window for enhancing neurotrophic effects of the targeted agent, such as Cerebrolysin. However, no clinical trials have been performed so far in the subacute stage of stroke investigating a potential synergistic effect of combining Cerebrolysin treatment with a standardized rehabilitation program.

The purpose of this study was to evaluate the efficacy of Cerebrolysin in terms of promoting additional motor recovery on top of a rehabilitation therapy during the subacute phase of stroke in patients with moderate to severe motor impairment. Evidence for the effects of Cerebrolysin on neuroplasticity has been investigated using functional neuroimaging.

## Methods

### Participants

Patients were included in the study within the first 7 days after stroke if they suffered from a first cortical, subcortical, or cortical-subcortical unilateral infarction confirmed by brain CT or MRI, with moderate to severe motor function involvement (total score of Fugl-Meyer assessment (FMA) 0–84) [12], had an inpatient status and were at the age between 18 and 80 years.

Exclusion criteria were progressive or unstable stroke, pre-existing and active major neurological disease or major psychiatric disease, a history of significant alcohol or drug abuse within the last 3 years, advanced liver, kidney, cardiac or pulmonary disease, a terminal medical diagnosis consistent with survival <1 year, substantial decrease in alertness at the time of randomization (score of ≥2 in National Institutes of Health Stroke Scale (NIHSS) item 1a), pregnancy or lactation, any condition contraindicated to Cerebrolysin including allergy to Cerebrolysin, participation in another stroke study, abnormal lab data or cardiopulmonary deficits interfering in physiotherapy, and a history of porcine brain peptide administration. Written informed consent was obtained from all subjects prior to inclusion in the study and the study protocol was approved by the Institutional Review Board (IRB) of each participating center (SMC IRB (2010-09-084, the leading ethics committee), Severance Hospital IRB (4-2012-0308), PNUYH IRB (02-2010-057), and CUH IRB (2010-10-154)).

### Experimental design

This IV trial was designed as a prospective, multicenter, randomized, double-blind, placebo-controlled, parallel-group study. The screening visit was performed within 7 days after stroke; demographic data, medical history, and data on physical examination and laboratory tests were documented. Enrolled patients were randomized to receive a 21-day treatment course (Days 8–28) of either Cerebrolysin or placebo, given as add-on to standardized rehabilitation therapy. Cerebrolysin was administered once daily at a dosage of 30 mL diluted with saline (total infusion solution 100 mL) by intravenous infusion over a time period of 30 min. Patients of the placebo group received 100 mL of saline instead. In addition, all patients received a standardized rehabilitation program consisting of 2 h of physical therapy and 1 h of occupational therapy daily on workdays (Monday to Friday). All patients in this study underwent the passive range of motion exercise in the patient’s room without comprehensive rehabilitation therapy before enrollment. After baseline assessment (Day 8; T0) efficacy and safety have been assessed immediately after treatment (Day 29; T1; study endpoint) as well as two (Day 60; T2) and three (Day 90; T3) months after stroke onset. The changes in neuroplasticity of the motor network were assessed by diffusion tensor imaging (DTI) and resting state functional magnetic resonance imaging (rsfMRI) at T0, T1, and T3. The study duration for each patient was 90 days.

### Stroke severity at baseline

Stroke severity at T0 was recorded using the NIHSS [13] in all enrolled patients. In addition, structural MRI data at T0 were used to assess initial lesion volumes of patients. The data were transformed to the same coordinate frame as the template brain, conforming to the Montreal Neurological Institute (MNI) space using the New Segment routine in Statistical Parametric Mapping (SPM)(http://www.fil.ion.ucl.ac.uk/spm/). Each patient’s lesion was manually delineated on the normalized structural image and then saved as a binary mask image. The number of voxels that composed the lesion mask was counted, and the volume of the lesion was measured by multiplying the voxel number by the voxel size.

### Motor function assessment

For motor function assessment, FMA was evaluated at baseline (T0), immediately after treatment (T1), two (T2) and three (T3) months after stroke onset. FMA scores were recorded separately for the upper limb (FMA-UL), lower limb (FMA-LL), and the total score (FMA-T). FMA has well-established reliability and validity as an indicator of motor impairment severity across different stroke recovery time points [14].

### Motor network plasticity assessment

The assessment of motor network plasticity was based on imaging data obtained from DTI and rsfMRI.

DTI data were collected using a 3 Tesla MR scanner. For every patient 46 whole brain images were acquired using a single-shot diffusion-weighted echo planar imaging sequence. The data set comprised 45 images with high diffusion weighting (b value = 1000 s/mm^2^) applied along 44 diffusion directions and one image with no diffusion weighting. Each image included 60 2.25-mm thick axial slices of 1.96 mm × 1.96 mm in-plane resolution. The data were preprocessed using the diffusion toolbox (FDT) included in the Functional Magnetic Resonance Imaging of the Brain’s (FMRIB) Software library (FSL) (http://fsl.fmrib.ox.ac.uk/fsl/) [15]. A diffusion tensor was modeled for each voxel, and fractional anisotropy (FA), axial diffusivity (AD), and radial diffusivity (RD) were computed from the diffusion tensor [16]. The maps of the DTI parameters in individual patients’ native space were transformed to the MNI space. As an alternative approach to tracking the corticospinal tract (CST) of every patient, a template CST acquired from healthy controls was used as a standardized approach to measure corticospinal integrity when using DTI data [17]. For generating the template CST, probabilistic tractography of the CST was performed for age-matched 23 healthy controls (mean age 53.5 ± 4.8 years). Tract-wise DTI parameters were calculated as the average of values read over the whole extent of the template CST, rather than over some regions of interest covering the partial extent of the template CST. For individual patients, CST-wise FA, AD, and RD were computed for the CST in the ipsilesional hemisphere (FA_ipsi_, AD_ipsi_, and RD_ipsi_).

Resting state functional magnetic resonance imaging (rsfMRI) data were collected using the same scanner as for DTI data. For every patient 100 whole brain images were acquired using a gradient echo planar imaging sequence (repetition time = 3000 ms, echo time = 35 ms). Each image included 35 4.00-mm thick axial slices of 1.72 mm × 1.72 mm in-plane resolution. The data were preprocessed using the routines in SPM (http://www.fil.ion.ucl.ac.uk/spm/) and Data Processing Assistant for Resting-State fMRI DPARSF (http://rfmri.org/DPARSF). Preprocessing steps included spatial realignment to the mean image, normalization to the same coordinate frame as the template brain conforming to the MNI space, spatial smoothing with a Gaussian kernel of 4 mm Full Width at Half Maximum (FWHM), removal of the systematic drift or trend, regressing out nuisance covariates such as the cerebrospinal fluid and white matter signals, and band-pass filtering at 0.01 to 0.08 Hz.

To estimate a sensorimotor network based on resting state functional connectivity, the representative time course from the primary motor cortex (M1) in the ipsilesional hemisphere served as a reference in determining correlation coefficients with all other time courses from the brain. The sensorimotor network for each group was displayed by thresholding a statistical parametric map of t-values calculated from a one sample *t*-test of individual patients’ sensorimotor networks, at an extent threshold of a *p*-value of 0.05 family-wise error corrected for multiple comparisons with a cluster-forming threshold of a *p*-value of 0.001. Furthermore, we computed a lateralization index (LI) between bilateral primary sensorimotor cortices (SM1s) by using the map of correlation coefficients to quantify a degree of symmetry of the sensorimotor network. For the voxels having values over the 95th percentile in the map, the LI was defined as the difference in the ratio of the voxels between ipsilesional and contralesional SM1s, such that LIs close to 0 referred to a symmetry in functional connectivity as exhibited in healthy individuals’ sensorimotor network.

### Safety analyses

A complete medical history and physical examination including vital signs were performed at screening. Laboratory data (hematology, blood chemistry, urinalysis) were assessed at all study visits from baseline (T0) to Day 90 (T3). All adverse events after giving informed consent have been documented and evaluated in terms of severity and causality.

### Statistical analysis

A sample size was determined a priori, which assumed a 2-tailed independent *t*-test with α equal to 0.05 and power at 80 %. The sample size was determined to be sufficient to detect a difference (δ) of 0.20 on the improvement of FMA-T from T0 to T1 as a primary outcome measure, with respective standard deviations of 0.27, as calculated from results of a similar previous study performed by our group [18]. Using the Lehr’s formula (16/(δ/σ)) [19] and a 15 % drop-out rate, it was calculated that 70 subjects in all would be needed.

In this study, the primary analysis was based on the intention to treat (ITT) population using the last observation carried forward method (LOCF), which included all randomized patients who received a least one dose of study medication and had a baseline and at least one post-baseline assessment of the primary endpoint (full analysis set). LOCF is characterized by individual missing values being replaced by the last observed value of that variable. The safety population included all patients who received at least one dose of study medication. Pre-planned stratified analyses for severe motor impairment at baseline (FMA-T <50) and moderate motor impairment at baseline (50≤ FMA-T ≤84) were performed in addition [20].

SPSS version 21.0 (SPSS, Chicago, IL, USA) was used for the statistical analyses. The difference in the continuous outcome between Cerebrolysin and placebo group was assessed using independent *t*-test. Frequency differences were tested using *χ*^2^-test or Fisher’s exact test. To test the effects of Cerebrolysin across all time points we used the repeated measures ANOVA with time as the within-patient factor and group (Cerebrolysin vs. placebo) as the between-patient factor for the parametric data with normal distribution. To correct for multiple comparisons made, a Bonferroni correction was used. A large F-value in the repeated measures ANOVA yields a correspondingly small *p*-value. The effect on FMA at T3 and its improvement from baseline was analyzed by a simple regression model with one independent variable by group. This analysis was performed to evaluate the pattern of motor function improvement at T3 in each group. *P*-values less than 0.05 were considered statistically significant.

## Results

**Fig. 1:**
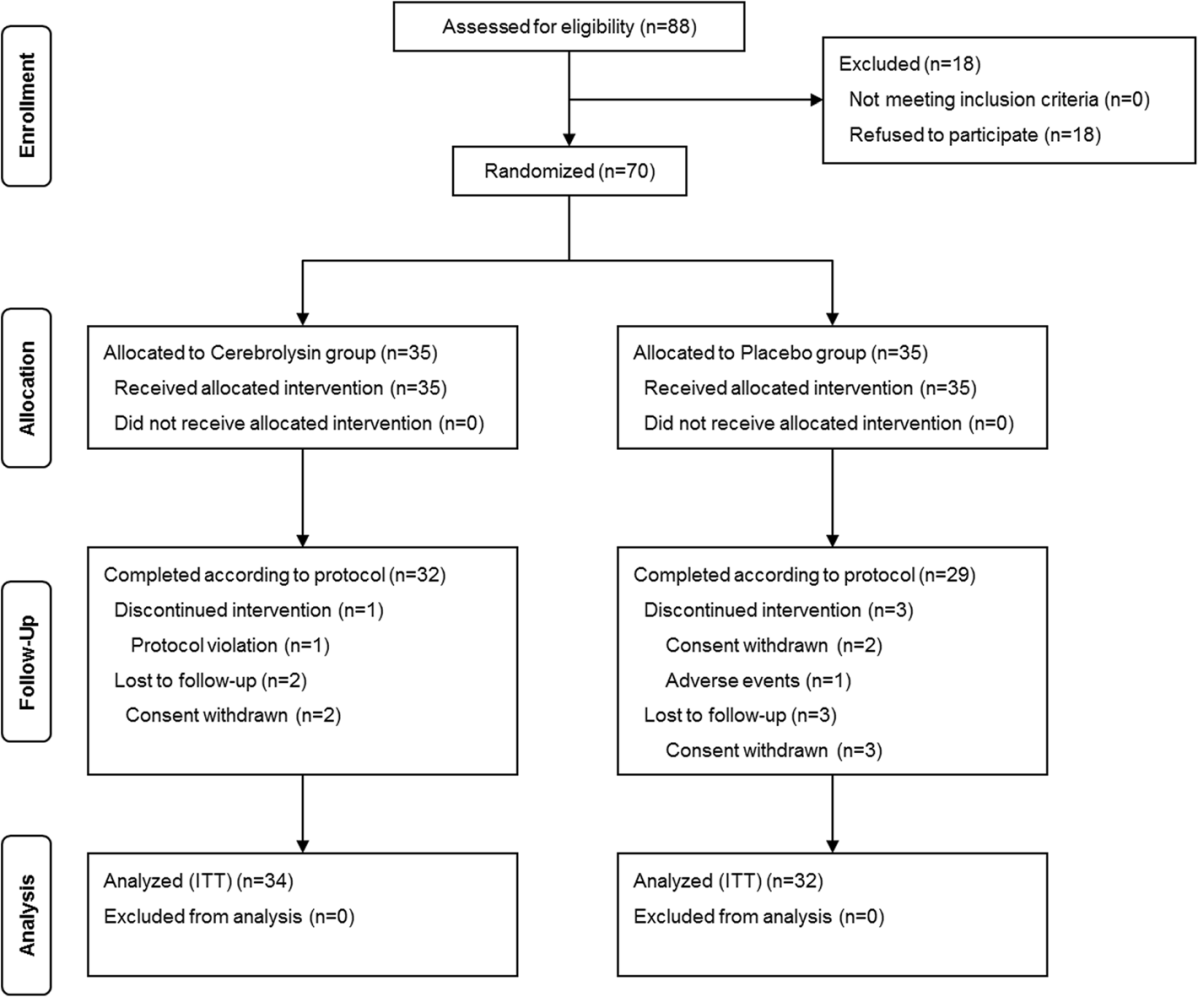
Enrollment and disposition of all patients participating in the clinical study. ITT, intention-to-treat

A total of 70 patients have been enrolled in this study (Fig. 1) from four study centers. All patients received at least one dosage of study medication (Cerebrolysin *n* = 35, placebo *n* = 35), thus representing the safety analysis set. A total of nine patients have discontinued study participation prematurely due to adverse events (hemorrhagic transformation; placebo *n* = 1), by withdrawing their consent (Cerebrolysin *n* = 2, placebo *n* = 5) or because of protocol violation (Cerebrolysin *n* = 1). A total of four patients (Cerebrolysin *n* = 1, placebo *n* = 3) had no post-baseline data and thus was excluded from the ITT analysis set (Cerebrolysin *n* = 34, placebo *n* = 32). The mean age of the patients was 64.2 ± 11.5 years, the proportion of males was 77.9 %, and the NIHSS mean was 7.6 ± 5.4. There were no relevant group differences from the ITT analysis set at baseline in general characteristics, stroke characteristics, or initial thrombolysis therapy (Table 1). There tended to be a higher proportion of patients with hypertension and arrhythmia in the Cerebrolysin group and relatively higher proportion of patients with hyperlipidemia, coronary artery disease and small vessel occlusion in the placebo group, but these differences are not statistically significant. There was no significant difference in NIHSS, lesion volume, and motor function measured by FMA at T0 between the two groups. In addition, there was no significant difference in presence of neglect in NIHSS at T0 between the two groups (Cerebrolysin *n* = 6, placebo *n* = 4). In subgroup analysis with severe motor impairment and moderate motor impairment at baseline, there was no significant difference in baseline characteristics between the Cerebrolysin and the placebo groups.

**Table 1:** Comparison of baseline characteristics (ITT analysis set)

Demographic parameter	Cerebrolysin (*n* = 34)	Placebo (*n* = 32)
Male sex: N (%)	29 (82.9)	24 (72.7)
Mean age: years (SD)	64.7 (10.1)	63.0 (10.6)
Mean weight: kg (SD)	65.4 (11.3)	66.7 (12.7)
Mean height: cm (SD)	165.7 (8.6)	165.7 (9.6)
Prevalence of risk factors: N (%)		
Hypertension	20 (57.1)	11 (33.3)
Hyperlipidemia	1 (2.9)	4 (12.1)
Diabetes mellitus	10 (28.6)	9 (27.3)
Arrhythmia	3 (8.6)	0 (0.0)
Coronary artery disease	3 (8.6)	4 (12.1)
Stroke side: N (%), Rt : Lt	14 (41.2) : 20 (58.8)	20 (62.5) : 12 (37.5)
Stroke etiology: N (%)		
Large artery atherosclerosis	13 (38.2)	11 (34.4)
Small vessel occlusion	10 (29.4)	14 (43.8)
Cardioembolism	7 (20.6)	4 (12.5)
Other determined	1 (2.9)	1 (3.1)
Undetermined ischemic stroke	3 (8.8)	2 (6.3)
Stroke lesion: N, cortical : subcortical : cortical-subcortical)	7: 6 : 21	8: 3 : 22
Stroke lesion characteristics		
Cortex	7	8
Cortex/BG/IC	3	0
Cortex/BG/IC/Corona radiata	3	1
Cortex/Corona radiata	0	2
BG/IC	9	14
BG/IC/Corona radiata	3	3
Corona radiata	8	3
Thalamus	1	2
Initial stroke treatment: N		
Intravenous thrombolysis	5	3
Intraarterial thrombolysis	1	2
Intraarterial thrombectomy	2	2
Baseline stroke severity: Mean (SD)		
NIHSS	8.4 (5.8)	7.0 (4.9)
Lesion volumes: cm^3^	15.560 (27.023)	19.253 (18.846)
Total FMA	42.0 (24.2)	42.2 (28.5)
Upper limb of FMA	24.6 (18.8)	26.7 (20.7)
Lower limb of FMA	17.4 (9.5)	15.5 (10.0)

*ITT* intention-to-treat, *BG* basal ganglia, *IC* internal capsule, *NIHSS* National Institutes of Health Stroke Scale, *FMA* Fugl-Meyer assessment

### Motor function assessment

**Fig. 2:**
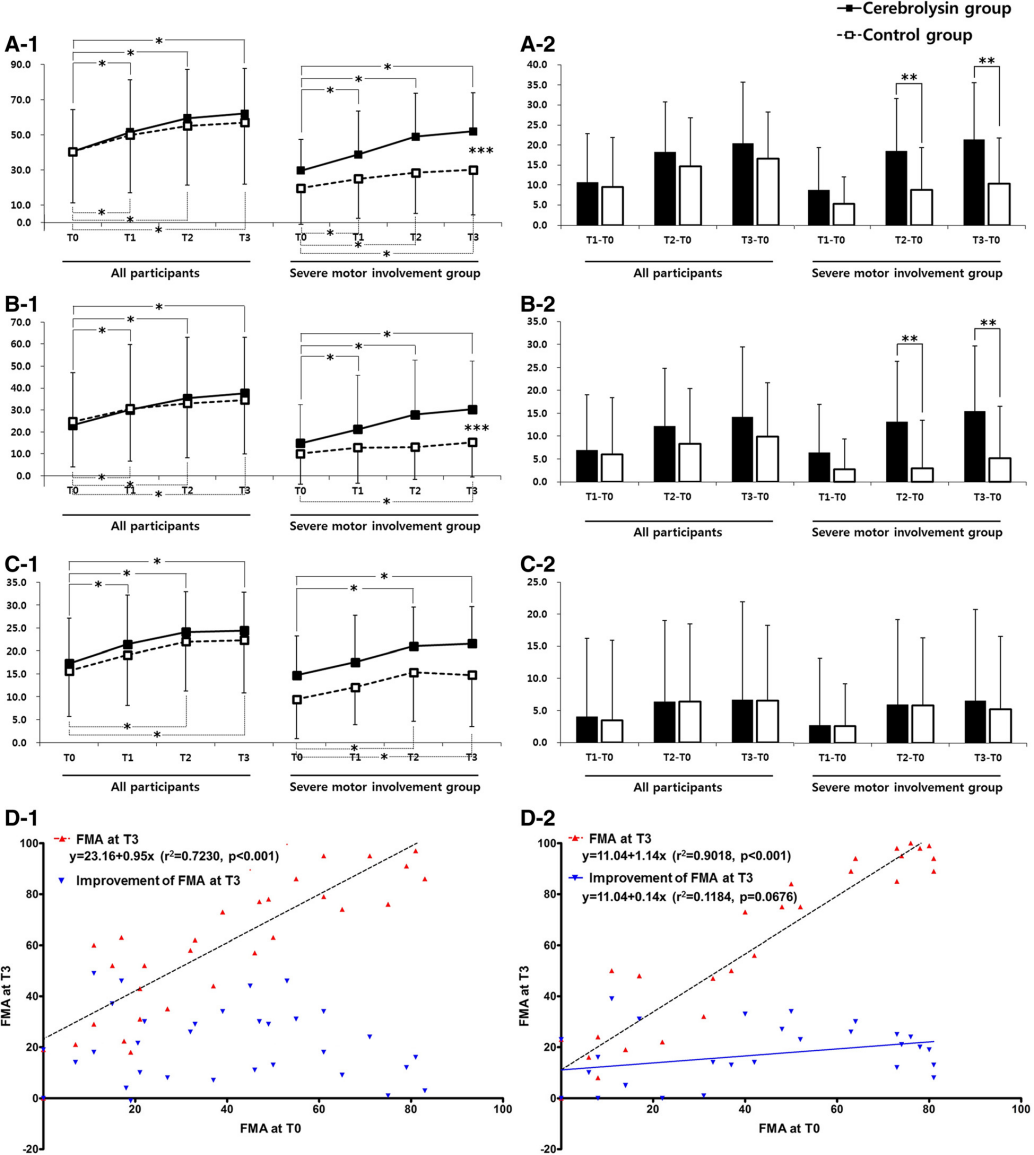
Changes in the Fugl-Meyer assessment (FMA) for Cerebrolysin (30 ml/day) and placebo at baseline (Day 8, T0), immediately after treatment (Day 29, T1) as well as two (Day 60, T2) and three (Day 90, T3) months after stroke onset. Analysis was based on the intention-to-treat (ITT) population using the last observation carried forward (LOCF) approach for missing data and the subgroup of patients with severe motor impairment (ITT-LOCF, FMA <50). The ITT-LOCF population included a total of 66 patients (Cerebrolysin *n* = 34, placebo *n* = 32), the subgroup a total of 37 patients (Cerebrolysin *n* = 20, placebo *n* = 17). Time courses (1) and improvements from baseline (2) are given for the total score of FMA (**a**), the upper limb subscore (**b**) and the lower limb subscore (**c**) for the total population (*left panel*) and the severe subgroup (*right panel*). **d** The simple regression analysis showed a significant relationship between FMA-T at T0 and T3 in both groups (*Red dots and broken lines*, Cerebrolysin group, **d-1**; placebo group, **d-2**). The improvement of FMA-T at T3 showed no relationship with baseline T0 scores in the Cerebrolysin group (**d-1**) (*Blue dots*), whereas the placebo group demonstrated a tendency of relationship between these two measures (**d-2**) (*Blue dots and line*). *p < 0.05 between time points in each group; **p < 0.05 between both groups; ***p < 0.05 between groups over time (repeated measures ANOVA)

In the ITT-LOCF analyses set both groups improved significantly over time in the FMA. However, repeated measures ANOVA showed no significant interaction effect between time and type of intervention as measured by FMA scores (FMA-T, FMA-UL, and FMA-LL). There were no significant differences in the improvement of FMA scores (FMA-T, FMA-UL, and FMA-LL) at T3 between the groups. The improvement of FMA-T and FMA-UL tended to be higher in the Cerebrolysin group than in the placebo group, but without statistical significance (Fig. 2).

In the ITT-LOCF subgroup analysis of patients with severe motor impairment on T0 (*n* = 37; Cerebrolysin *n* = 20, placebo *n* = 17; FMA-T at T0 <50), repeated measures ANOVA showed a significant interaction effect between time and type of intervention as measured by FMA-T (F_3,102_ = 4.596, *p* < 0.05)(Fig. 2a-1 right panel) and FMA-UL (F_3,102_ = 3.605, *p* < 0.05)(Fig. 2b-1 right panel). In addition, there was a significant group difference in the FMA-T (Fig. 2a-2 right panel) and FMA-UL (Fig. 2b-2 right panel) at T2 and T3. The simple regression analysis showed a relationship between FMA-T at T0 and T3 in both groups (Cerebrolysin *r*^2^ = 0.7230, *p* < 0.001, Fig. 2d-1 and placebo *r*^2^ = 0.9018, *p* < 0.001, Fig. 2d-2). However, the improvement of FMA-T at T3 showed no relationship with baseline scores (T0) in the Cerebrolysin group (*r*^2^ = 0.0086, *p* = 0.6137; Fig. 2d-1), whereas the placebo group demonstrated a tendency of relationship between these two measures (*r*^2^ = 0.1184, *p* = 0.0676, Fig. 2d-2).

In the ITT-LOCF subgroup analysis of patients with moderate motor impairment on T0 (*n* = 29; Cerebrolysin *n* = 14, placebo *n* = 15; 50≤ FMA-T at T0 ≤84), repeated measures ANOVA showed no significant interaction effect between time and type of intervention as measured by FMA scores (FMA-T, FMA-UL, and FMA-LL).

### Motor network plasticity assessment

**Fig. 3:**
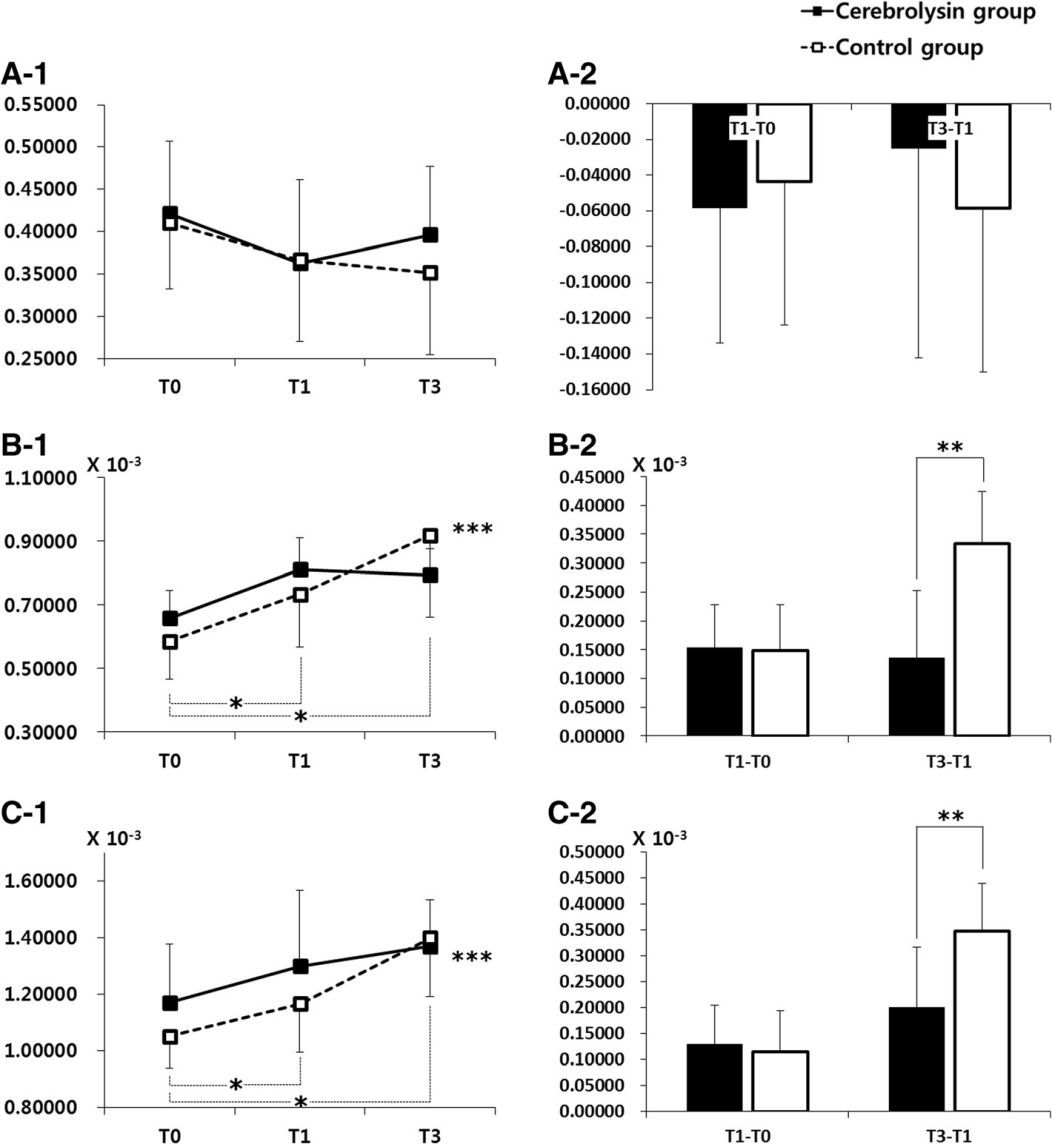
Changes in the diffusion tensor imaging (DTI) for Cerebrolysin (30 ml/day) and placebo at baseline (Day 8, T0), immediately after treatment (Day 29, T1) and three (Day 90, T3) months after stroke onset. Analysis was based on the intention-to-treat (ITT) population using the last observation carried forward (LOCF) approach for missing data in the subgroup of patients with severe motor impairment (FMA <50). Time courses (1) and changes from baseline (2) are given for the fractional anisotropy (FA; **a**), the axial diffusivity (AD; **b**), and the radial diffusivity (RD; **c**). *p < 0.05 between time points in each group; **p < 0.05 between both groups; ***p < 0.05 between groups over time (repeated measures ANOVA)

In DTI analysis of the CST based on the ITT-LOCF subgroup analysis of patients with severe motor impairment on T0, repeated measures ANOVA showed significant interactions between time and type of intervention for AD_ipsi_ (F_2,59_ = 2.831, *p* < 0.05, Fig. 3b-1) and RD_ipsi_ (F_2,59_ = 3.490, *p* < 0.05, Fig. 3c-1). Moreover, there were significant differences between the two groups in the changes of AD_ipsi_ and RD_ipsi_ at T3 (*p* < 0.05) (Fig. 3b-2, C-2). For FA_ipsi_, however, repeated measures ANOVA showed no significant interaction between time and type of intervention (Fig. 3a-1, A-2). In the ITT-LOCF subgroup analysis of patients with moderate motor impairment on T0, repeated measures ANOVA showed no significant interaction effect between time and type of intervention as measured by DTI (FA_ipsi_, AD_ipsi_ and RD_ipsi_).

**Fig. 4:**
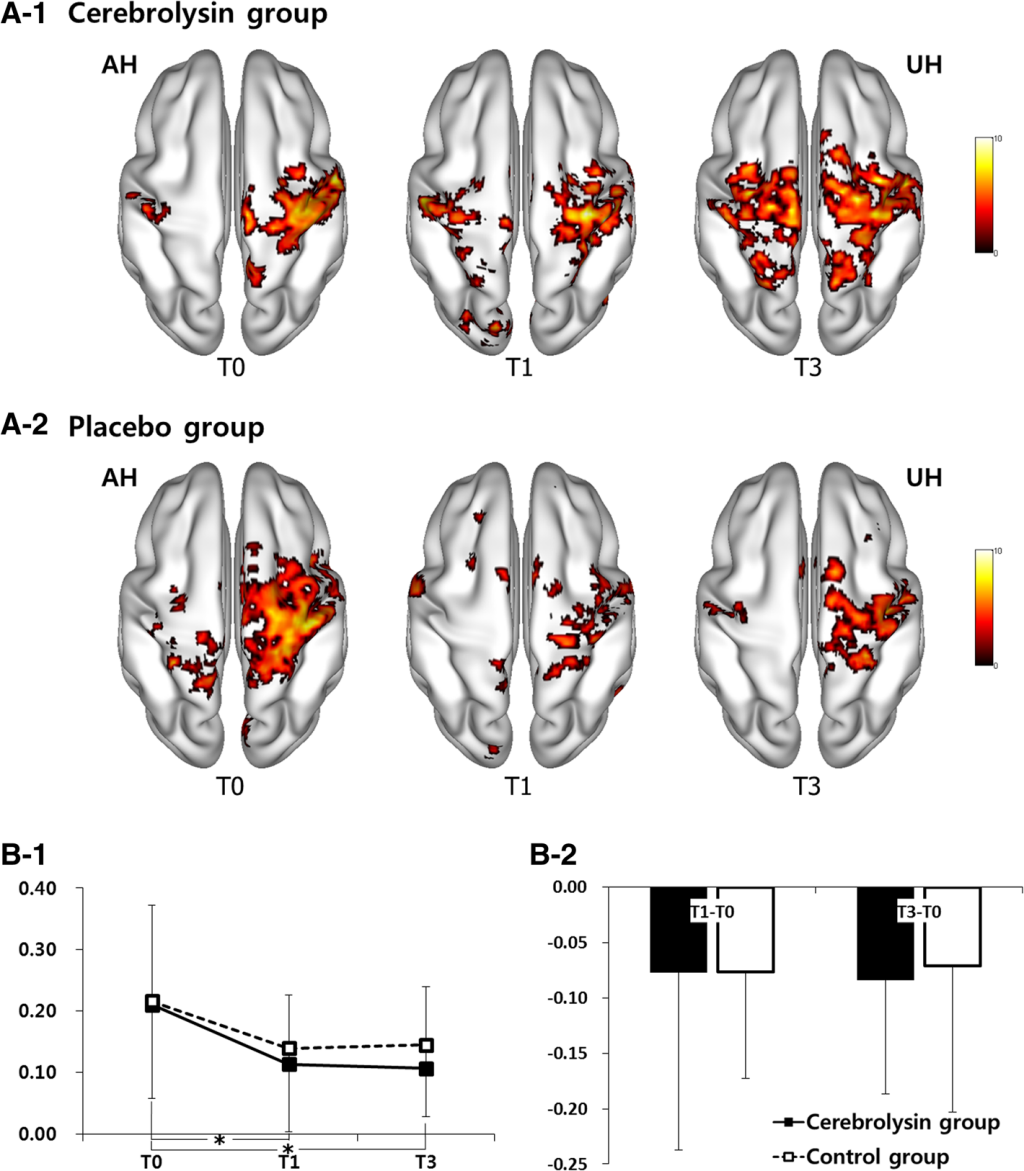
Resting state of the sensorimotor network as shown by the resting state functional MRI (rsfMRI) for Cerebrolysin (30 ml/day; **a1**) and placebo (**a2**) in the affected (AH) and unaffected (UH) hemispheres at baseline (Day 8, T0), immediately after treatment (Day 29, T1) and three (Day 90, T3) months after stroke onset. Analysis was based on the intention-to-treat (ITT) population using the last observation carried forward (LOCF) approach for missing data in the subgroup of patients with severe motor impairment (FMA <50) from 29 patients (Cerebrolysin *n* = 13, placebo *n* = 16). Time course (**b1**) and changes from baseline (**b2**) are given for the lateralization index. *p < 0.05 between time points in each group

Among the ITT-LOCF subgroup analysis of patients with severe motor impairment on T0, rsfMRI data were analyzed from 29 patients (Cerebrolysin *n* = 13, placebo *n* = 16). Changes in the sensorimotor network across time showed increased symmetric functional connectivity between the bilateral primary sensori-motor cortices (SM1s) specifically in the Cerebrolysin group (Fig. 4a-1, a-2). Indeed, although repeated measures ANOVA showed no significant interaction between time and type of intervention in the analysis of the lateralization index (LI) between bilateral SM1s, (Fig. 4b-2), only Cerebrolysin showed a significant difference in the change of the LI at T1 and T3 (Fig. 4b-1).

### Safety analyses

Of all patients treated a total of 94.3 % received 21 infusions (Cerebrolysin 97.1 %, placebo 91.4 %). In each study group one patient suffered from a serious adverse event (SAE), none of both SAEs was rated as related to study medication. The SAE in the Cerebrolysin group was described as cholecystitis with gallstone, which resolved during the study period. The SAE in the placebo group was a hemorrhagic transformation of the cerebral infarction, the patient discontinued study participation due to this event. None of the patients died during the study. Vital signs and laboratory values were similar between treatment groups and did not show clinically relevant changes during the course of the study.

## Discussion

The goal of this study was to investigate whether a 3 weeks treatment with Cerebrolysin in the subacute phase of stroke given on top of a standardized rehabilitation therapy provides additional benefit on motor recovery in patients with moderate to severe motor impairment. The results of this study revealed that Cerebrolysin treatment for 3 weeks in the subacute phase of stroke, in addition to rehabilitation therapy, tended to show better improvement of motor function at 3 months after stroke onset than was seen in the placebo group, but without statistical significance. However, in patients with severe motor involvement at 7 days after stroke onset, Cerebrolysin as an additional treatment with a standardized rehabilitation showed significant better improvement of motor function at 3 months after stroke onset. Also, Cerebrolysin treatment for 3 weeks during the subacute phase of stroke showed no serious adverse effects. The combination of standard rehabilitation therapy with Cerebrolysin treatment in the subacute stroke has shown additional benefit on motor recovery in patients with severe motor impairment.

The optimal timing for rehabilitation is still under discussion but there is evidence that an earlier start of rehabilitation might be more effective [21]. In fact, the therapeutic time window for functional recovery seems to be relatively broad from days to weeks [22]. Functional recovery refers to enhanced sensory and motor performance after stroke and might also include a varying degree of behavioral compensation [21]. However, pure recovery is based on the neuroplasticity and takes advantage of the diffuse and redundant connectivity existing in the brain and the remapping between related cortical regions in order to form new structural and functional circuits [21]. Animal studies have shown similarities in plasticity-relevant gene expression and translation between early brain development and the semi-acute phase after stroke [21]. These genes and proteins are important for neuronal growth, synaptogenesis and the proliferation of dendritic spines. Previous in vivo and in vitro studies have shown similar effects on neurons by Cerebrolysin [23–29]. In animal stroke studies [28, 30] rehabilitation of motor-sensory function was significantly increased when Cerebrolysin administration was initiated within 48 h after stroke, however, in humans the time window for recovery is expected to be longer or even never really closing but plastic processes diminish and slow with time [21].

This study could not achieve the primary objective to evaluate the efficacy of Cerebrolysin for motor recovery measured the improvement of FMA-T from baseline to immediate after treatment in patients with moderate to severe motor impairment. However, a combination of a standardized rehabilitation program with Cerebrolysin treatment was more effective on the improvement of severe motor deficits at 3 months after stroke onset as compared to a combination with placebo. In addition, regression analysis has shown that the magnitude of improvement in motor functions by Cerebrolysin was independent of baseline severity, which was reflected by a faster and more pronounced motor improvement in patients with more severe motor impairment at baseline as compared to placebo. This improvement might be considered as enhancement of motor control function mainly in the upper extremities rather than in the lower extremities as indicated by the separate analyses of upper and lower extremities. No significant difference in the improvement of motor function at immediately after treatment may be due to relative small number of patients with severe motor involvement. This constitutes one of the limitations of the present study. Further studies with a larger sample size will be needed to elucidate this issue. A reason for failure of the primary objective might be the reported the possible ceiling effect on hand and lower extremity items [31]. Another reason might be therapeutic potentials of stroke rehabilitation. The additional effect of Cerebrolysin could be hidden due to the conventional rehabilitation strategies in the subacute stroke patients with moderate motor impairment. On the other hand, the additional effect of Cerebrolysin treatment in the subacute stroke stage could have important implications for stroke rehabilitation, because the conventional rehabilitation strategies are somewhat limited in their improvement of motor function in stroke patients with severe motor involvement [2].

In subgroup of patients with severe motor impairment at baseline, we additionally investigated the effect of Cerebrolysin on motor network plasticity with DTI and rsfMRI, which has been done for the very first time in a Cerebrolysin study. CST-wise DTI analysis has shown significant interactions for both RD and AD between intervention types and time in such a way that increases in the diffusivity across time were less steep or restricted for the Cerebrolysin group. RD was shown to increase after injury reflecting demyelination [32, 33] and thus, a restriction of RD increments for the Cerebrolysin group may suggest that Cerebrolysin plays a role in the protection against demyelination of the CST during the subacute phase of stroke. On the other hand, a decrease in AD may indicate axonal damage in the acute phase after injury, whereas an elevation of AD may occur due to degenerative processes in the chronic phase [33]. Interpretation by connecting directional diffusivities to discrete pathological processes is still controversial, but steeper increases in AD as seen in the placebo group might reflect a composite of degeneration and subsequent structural compensation that does not necessarily yield functionally meaningful connections [34]. In addition, although no interaction between intervention types and time was shown for FA, it is notable that FA started to increase after T1 in the Cerebrolysin group, whereas it continuously decreased until T3 in the placebo group. This may reflect recovery of corticospinal integrity promoted by the pharmacological action of Cerebrolysin. While CST-wise DTI parameters exhibit changes in the motor-related white matter, the LI between bilateral SM1s in the resting state sensorimotor network shows changes in the motor-related cortical grey matter [35]. In the Cerebrolysin group symmetric functional connectivity was more pronounced indicating a better recovery of motor cortical function.

This study possesses some limitations. Even though the baseline motor function is the most important prognostic factor, there are numerous other factors in motor recovery in stroke patients, such as cognitive function, aphasia, comorbid medical conditions, stroke-related complications, socioeconomic status, and extent of family and social support [2, 36–39]. In this study, a complete assessment at baseline could not be performed for all potential prognostic factors for motor recovery in stroke patients. The relatively small number of patients enrolled in this study could not allow multivariate models to adjust for more confounding variables. Therefore, further study with a larger number of participants and long-term follow-up would be necessary to better evaluate the effect of Cerebrolysin in combination with rehabilitation on recovery in subacute stroke patients. There was no significant difference in intensity and duration of rehabilitation therapy after treatment (T1) between the two groups. However, participants could not be regulated for rehabilitation therapy from after treatment (T1) to 3 months after stroke onset (T3), although any other neuroprotective or nootropic drug was not allowed until 3 months after stroke onset. Stroke severity and age of stroke patients in this study were relatively low compared with other stroke trials. These might be due to many strict exclusion criteria on the screening visit within 7 days after stroke. For the motor network plasticity assessment in this study, we used imaging data obtained from DTI and rsfMRI that have been successfully applied in patients with acute and chronic stroke to evaluate motor network [40, 41]. However, rsfMRI in stroke patients has certain limitations as highly susceptible to motion-related artifacts because patients tend to move more than controls [40]. Due to these limitations, we could not analyze the rsfMRI data of some participants. Especially, the number of patients who provided analyzable rsfMRI data set for three time points were limited in patients with moderate motor impairment; ten in the Cerebrolysin group and only three in the placebo group. Since these numbers were too small to get a reliable group-level analysis, we did not address group results for patients with moderate motor impairment. Therefore, additional study will be needed to elucidate these issues. In spite of favorable outcomes of Cerebrolysin in acute stroke patients [8], a recent review [42] did not demonstrate clinical benefits of Cerebrolysin for treating acute ischemic stroke, and recommended that further well-designed randomized controlled trials would be required to obtain a better understanding of the potential value or risks of Cerebrolysin in acute ischemic stroke.

## Conclusions

This study has shown that Cerebrolysin treatment over 3 weeks in combination with rehabilitation therapy in the subacute phase of stroke is safe and provides a beneficial effect on motor recovery in patients with severe motor impairment. Furthermore, for the very first time neuroimaging data have shown that treatment with Cerebrolysin had a beneficial influence on both, motor-related grey and white matter. Further studies also with a larger sample size will be needed to clarify the impact and the appropriate time window for Cerebrolysin treatment in order to optimize motor recovery after ischemic stroke by enhancing motor network plasticity. Also, there were no safety concerns with Cerebrolysin. From this study, Cerebrolysin treatment as add-on to a rehabilitation program might be considered as a pharmacologic approach for motor recovery in ischemic stroke patients with severe motor involvement in the subacute stage.
